# Characterizing deaths among adult patients with severe acute respiratory infection: during the pre- and COVID-19 pandemic periods in Bangladesh, 2018–2022

**DOI:** 10.1186/s41182-023-00565-1

**Published:** 2023-12-19

**Authors:** Md Zakiul Hassan, Md. Ariful Islam, Homayra Rahman Shoshi, Md. Kamal Hossain, Tahmina Shirin, Fahmida Chowdhury

**Affiliations:** 1 Programme for Emerging Infections, Infectious Disease Division, International Centre for Diarrhoeal Disease Research, Bangladesh (Icddr,b), Mohakhali, Dhaka, Bangladesh; 2 Institute of Epidemiology, Disease Control and Research (IEDCR), Dhaka, Bangladesh

**Keywords:** SARI, Pandemic, Influenza, SARS-CoV-2, In-hospital deaths, Post-discharge deaths

## Abstract

**Background:**

Severe acute respiratory infection (SARI) is a leading cause of mortality globally, peaking during the COVID-19 pandemic. We analyzed SARI-associated deaths during the pre-and-pandemic periods in Bangladesh to identify the contributing factors.

**Methods:**

We analyzed data from hospital-based influenza surveillance at nine tertiary-level hospitals in Bangladesh. We considered March 2018–February 2020 as the pre-pandemic period and March 2020–February 2022 as the pandemic period and included adult (≥ 18 years) participants in our study. Surveillance physicians identified WHO-SARI case definition meeting inpatients and collected demographics, clinical characteristics, and outcomes at hospital discharge and 30 days post-discharge. We performed rRT-PCR for influenza and SARS-CoV-2 viruses on collected nasopharyngeal and oropharyngeal swabs. We used multivariable Cox's regression models to calculate the hazard ratio (HR) for factors associated with SARI deaths in these adult patients.

**Results:**

We enrolled 4392 SARI patients during the pre-pandemic and 3824 SARI patients during the pandemic period. Case fatality ratio was higher during the pandemic: 13.62% (521) [in-hospital: 6.45% (247); post-discharge: 7.17% (274)] compared to pre-pandemic, 6.01% (264) [in-hospital: 2.01% (89), post-discharge: 4% (175)] (p < 0.001). Pre-pandemic, influenza was detected in 14% (37/264) of SARI deaths. Influenza was detected during the pandemic in 2.3% (12/521), SARS-CoV-2 in 41.8% (218/521), and both viruses in only one SARI death. History of smoking and the presence of 1 or more co-morbid conditions independently attributed to SARI deaths in adults in the pre-pandemic period. SARI deaths in such patients were also associated with respiratory difficulties on admission in both pre-pandemic (aHR 2.36; 95% CI:1.65–3.36) and pandemic period (aHR 2.30; 95% CI: 1.57–3.35) after accounting for age, sex, smoking status, presence of 1 or more co-morbid conditions, and detection of influenza and SARS-CoV-2 viruses.

**Conclusions:**

During the pandemic, SARI mortality increased; influenza-associated mortality declined, and SARS-CoV-2 caused over a third of SARI deaths. Post-discharge mortality was higher than in-hospital mortality during both periods. Limiting premature discharge and strengthening post-discharge monitoring and nursing services could reduce unexpected deaths. Formative research to better understand post-discharge mortality is essential to reduce SARI deaths.

**Supplementary Information:**

The online version contains supplementary material available at 10.1186/s41182-023-00565-1.

## Background

Severe acute respiratory infections (SARI) have long been a formidable threat to global public health, causing significant morbidity and mortality across all age groups [1]. The primary contributors to SARI mortality are viruses, with influenza, respiratory syncytial virus, rhinovirus, and human metapneumovirus among the major causative pathogens [2–4]. Each year, SARI leads to an alarming 4.2 million deaths globally, with children under five being particularly vulnerable, accounting for 1.4 million deaths [5–7]. Influenza alone infects 5–15% of people and causes an estimated 291,000 to 650,000 annual deaths worldwide [8].

Low- and middle-income countries (LMICs) bear a substantial burden of SARI-related mortality, with nearly 90% of SARI-associated deaths occurring in these regions [9]. In Bangladesh, the influenza season during the monsoon is responsible for thousands of SARI deaths linked to influenza, highlighting the need for preventive and control efforts [10, 11]. Additionally, SARI is often seen in conjunction with co-morbid conditions like cardiovascular disease, tuberculosis, and Human Immunodeficiency Virus (HIV) infection, further exacerbating the mortality risk [12–15].

The emergence of the corona virus disease 2019 (COVID-19) pandemic caused by Severe Acute Respiratory Syndrome Corona Virus 2 (SARS-CoV-2) further intensified the challenges posed by SARI, resulting in 6.9 million deaths worldwide [16]. During this period, SARI cases caused by SARS-CoV-2 surged, with a notable increase in in-hospital deaths and 30-day post-discharge mortality rates [17]. The pandemic revealed that SARI mortality was no longer solely attributed to influenza, but also heavily impacted by COVID-19. Countries like Brazil and India experienced a dramatic rise in SARI cases and deaths during the pandemic period [18, 19].

Understanding the factors affecting SARI mortality is vital to develop effective control measures. Age and pre-existing medical conditions, such as chronic heart disease, chronic obstructive pulmonary disease (COPD), asthma, and HIV infection, significantly contribute to unfavorable outcomes in SARI patients [20–22]. However, there are critical data gaps from LMIC, such as Bangladesh, particularly in looking into the impact of other contributing factors leading to SARI deaths during the pandemic period.

This study aims to comprehensively characterize deaths among SARI patients during the pre- and COVID-19 pandemic periods in Bangladesh. By investigating the drivers of SARI mortality, we can develop targeted control strategies, strengthen surveillance efforts, and formulate vaccination policies to effectively combat SARI-mortality.

## Methods

### Study settings and study population

**Fig. 1:**
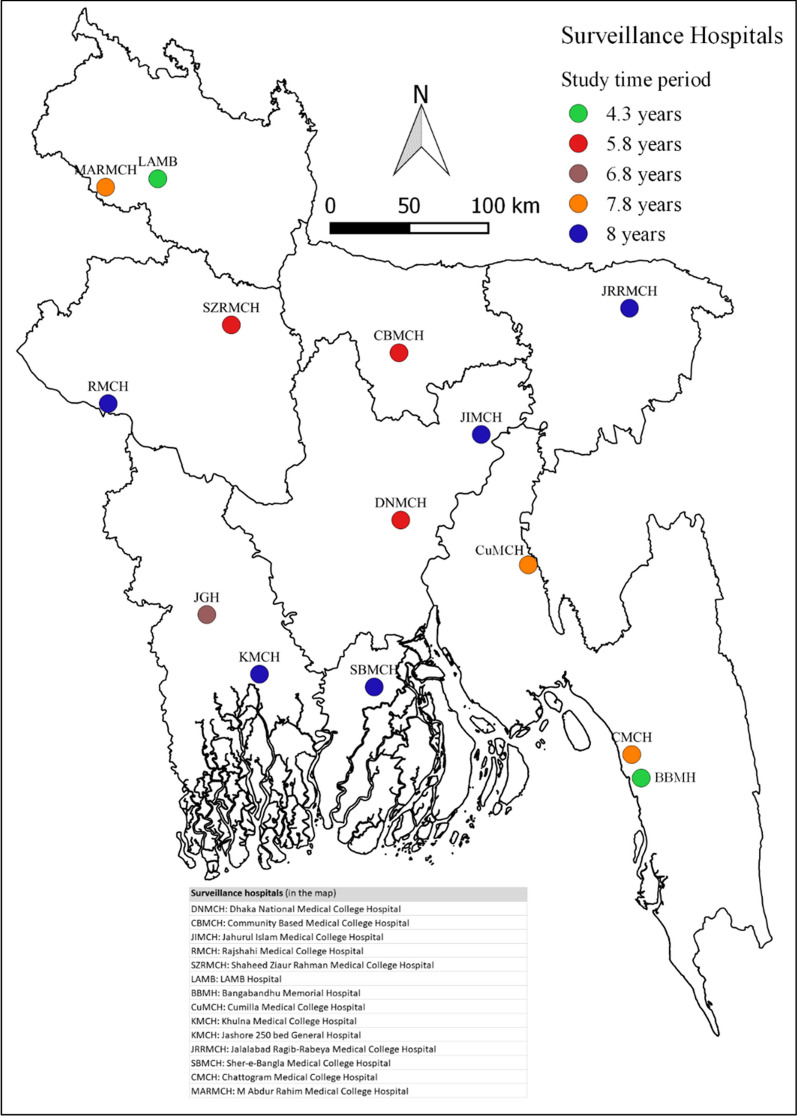
Location of the hospitals included in the Hospital-Based Influenza Surveillance in Bangladesh

We analyzed data from our Hospital-Based Influenza Surveillance (HBIS) to characterize both in-hospital and post-discharge SARI deaths. The HBIS was initiated in 2007 and currently ongoing in nine tertiary care level hospitals (seven public and two private) geographically distributed all over Bangladesh (Fig. 1). The activities of the surveillance system is carried out jointly by the International Centre for Diarrhoeal Disease Research, Bangladesh (icddr,b) and the Institute of Epidemiology, Disease Control and Research (IEDCR), of the Government of Bangladesh (GoB) with the technical support from the United States Centers for Disease Control and Prevention (US CDC). The in-patient capacity of the surveillance hospitals ranges from 500 to 1500 beds with a 100%–150% bed occupancy rate.

For this study, we analyzed data for the adult SARI patients (aged 18 years or more) enrolled from March 2018 to February 2022. Despite the ongoing pandemic in 2020 with subsequent pandemic control efforts, the surveillance remained active and continued its operations 6 days a week (Saturday to Thursday) by collecting data from the in-patient departments of the study hospitals. In this study, the pre-COVID-19 pandemic period spanned from March 2018 to February 2020, and the COVID-19 pandemic period was considered from March 2020 to February 2022.

### Case definition of severe acute respiratory infections (SARI)

SARI was defined according to WHO case definition “as an acute respiratory infection with history of fever or measured fever of ≥ 38 C° and cough with onset within the last 10 days and requires hospitalization”.

### Case identification, data collection, and post-discharge follow-up

The study physicians and support staff of this study screened and identified SARI patients meeting the SARI case definition from in-patient departments of medicine wards, coronary care units (CCU), and specialized COVID-19 isolation wards established during the COVID-19 pandemic period.

After identifying adult SARI patients, written informed consent was obtained from them. The study physicians then enrolled and performed a physical examination of all the participants. This was followed by collection of data using a standardized surveillance data collection form in a handheld computer. The form included demographic, clinical, and available diagnostic findings of the patients. At the time of discharge, the outcome status (full recovery, partial recovery, referral to another facility, and in-hospital death) of the participants were recorded. Each discharged patient/their family members was then contacted over phone again after 30 days of discharge to assess their post-discharge status. In case a participant died after hospital discharge, information regarding the date, place and probable cause of death was collected from their family members. This report only includes deaths that were caused by the patient's underlying medical condition. Post-discharge accidental deaths were excluded.

### Sample collection and laboratory analysis

Maintaining all aseptic precautions, nasopharyngeal and oropharyngeal swabs were collected from the SARI patients who gave written informed consent to participate in this study. Swabs were placed into viral transport media, stored in a liquid nitrogen dry shipper on site and transported to the virology laboratory of icddr,b from each site every week. All the collected samples were tested for the RNA of influenza virus by real time reverse transcription-PCR (rRT-PCR) during the pre-pandemic period and for both the influenza and SARS-CoV-2 virus during the pandemic period. Since November 2021, we have been using influenza SARS-CoV-2 (Flu SC2) multiplex assay to simultaneously detect influenza and SARS-CoV-2 virus.

### Data analysis

We analyzed categorical variables using frequency and percentages and compared the percentage using two sample z-tests. We analyzed numerical variables using the median and inter-quartile range (IQR) and compared groups using nonparametric equality-of-medians tests. We calculated the proportion of SARI cases that died during the pre- and COVID-19 pandemic periods and compared to the proportion who were alive during these periods, excluding accidental deaths. Potential risk factors for SARI deaths, including age, sex, smoking history, difficulty breathing at any time during hospitalization, ICU admission, length of hospital stay in days, duration of symptoms prior to admission in days, presence of at least one co-morbid conditions, reported during hospitalization and the detection of influenza or SARS-CoV-2 viruses (only during the pandemic period) were assessed by comparing patients who died during the pre- and pandemic period with survivors. The co-morbid conditions included in this analysis were the presence of asthma, COPD, diabetes, hypertension, heart disease, lung disease, chronic kidney disease, chronic liver disease, HIV/AIDS, malaria and cancer in SARI patients.

Based on the literature and experience [23], we classified the variables into four levels. The first and most distal level variables included sex and age (Additional file 1: Fig. S1). The second hierarchical level variable was smoking history. The third hierarchical level variables included the presence of at least one co-morbid condition and the detection of influenza in the pre-pandemic period and influenza /SARS-CoV-2 in the pandemic period. The fourth and most proximal level variables included difficulty breathing, length of hospital stay, duration of symptoms before hospital admission, and ICU admission. All the variables were considered categorical variables apart from age, duration of symptoms before hospitalization and length of hospital stay which were considered continuous variables. We estimated hazard ratios with 95% confidence intervals using multivariable Cox regression model.

At first, all the explanatory variables (potential risk factors) of each level were entered into their respective models. Then we identified initial risk factors at a 5% level of significance at each level. For each level, selected initial risk factors were jointly included in the model, controlling for all variables retained in previous levels with a 10% level of significance. Then, progressively excluded variables at each level, retaining only those variables that were statistically significant at a 5% level of significance. Therefore, in the final model, hazard ratios were simultaneously adjusted for variables in the same hierarchical level and those retained in previous levels. All analyses were conducted with Stata 13.0 software (StataCorp. 2013. Stata Statistical Software: Release 15. College Station, TX: StataCorp LP).

### Ethical clearance

Study participants provided informed written consent. The institutional review board of icddr,b reviewed and approved the study protocol.

## Results

### Demographics of the study patients

During the pre-pandemic period, we enrolled 4392 adult SARI patients with the median age 45 years (IQR: 28–60 years), and of them 63% was male (Table 1). Among these patients, 264 (6.01%) died during the study period. Of these deaths, 89 (2.01%) occurred during hospitalization, and 175 (4%) occurred within 30 days post-discharge. During COVID-19 pandemic period, through this surveillance platform we enrolled 3824 adult SARI patients. Median age of the enrolled patients was 50 years (IQR: 35–60 years), with 61% being male. During this pandemic period, 521 (13.62%) SARI patients died. Of these, 247 (6.45%) died in the hospital, while 274 (7.17%) died within 30 days of post-discharge period. Approximately 17% of our participants were vaccinated with at least a single dose of vaccine against SARS-CoV-2 during this period. The B1.1.7 (alpha) variant of SARS-CoV-2 was predominant till December 2020 in Bangladesh, which was later followed by B.1.617.2 (delta) variant and B1.351 (beta) variant of SARS-CoV-2.

**Table 1:** Clinico-epidemiological characteristics of SARI patients during the pre- and COVID-19 pandemic periods

	Patient enrollment		
	Total *N* = 8216	Pre-COVID-19 pandemic, *N* = 4392	During COVID-19 pandemic, *N* = 3824
	*n* (%)	*n* (%)	*n* (%)
Demographic characteristics			
Age			
18–30 years	1688 (20.55)	1153 (26.25)	535 (13.99)
31–40 years	1133 (13.79)	565 (12.86)	568 (14.85)
41–50 years	1228 (14.95)	579 (13.18)	649 (16.97)
51–60 years	1486 (18.09)	731 (16.64)	755 (19.74)
≥ 60 years	2681 (32.63)	1364 (31.06)	1317 (34.44)
Median age (IQR), years	50 (32–60)	45 (28–60)	50 (35–60)
Male	5127 (62.40)	2783 (63)	2344 (61)
Clinical characteristics			
Runny nose	3186 (38.78)	1744 (39.71)	1442 (37.71)
Headache	4597 (56)	2690 (61.25)	1907 (49.87)
Sore throat	1736 (21.13)	993 (22.61)	743 (19.43)
Difficulty breathing reported on admission	5542 (67.45)	2491 (56.72)	3051 (79.79)
Body ache	4420 (53.80)	2474 (56.33)	1946 (50.89)
≥ 1 co-morbid condition (Self-reported)	3568 (43.43)	1760 (40)	1808 (47.28)
Duration of symptoms prior to admission in days; median (IQR)	4 (3–6)	4 (2–5)	4 (3–6)
Length of hospital stay in days; median (IQR)	3 (2–5)	3 (2–5)	3 (2–6)
Treatment received			
Antibiotic	6847 (83.34)	3257 (74.16)	3590 (93.88)
Oxygen	3982 (48.54)	1248 (28.50)	2734 (71.51)
Mechanical ventilation	23 (0.28)	17 (0.40)	6 (0.16)
ICU support	28 (0.34)	19 (0.43)	9 (0.24)
Laboratory results			
SARS-CoV-2 detected (*n* = 3816) ^$^	1043 (27.33)	0 (0)	1043 (27.33)
Influenza virus detected	1312 (16)	1010 (23)	294 (7.70)
Influenza virus types			
Influenza A	919 (70.05)	682 (67.52)	237 (78.48)
Influenza B	393 (29.95)	328 (32.48)	65 (21.52)
Influenza A and B	0 (0)	0 (0)	0 (0)
Subtypes of influenza A virus			
Influenza A(H1N1) pdm09	571 (43.52)	409 (40.50)	162 (53.64)
Influenza A(H3N2)	339 (25.84)	264 (26.14)	75 (24.83)
Influenza A(H5N1)	0 (0)	0 (0)	0 (0)
Influenza A (inconclusive)^Ȳ^	9 (0.7)	9 (0.89)	1 (0.2)
Clinical outcome			
Death (total)	785 (9.55)	264 (6.01)	521 (13.62)

^$^Testing of SARS-CoV-2 was initiated in March 2020

^Ȳ^Inconclusive: unable to characterize due to insufficient viral load in samples

### Viral etiology of SARI deaths pre- and during the COVID-19 pandemic

We tested all SARI decedents (both in-hospital and post-discharge deaths) for the influenza virus during the pre-pandemic period. Among the 264 decedents, influenza virus was identified in 14% (37) of cases. Of the 264 decedents, 18% of influenza-positive SARI patients died during hospitalization, and 12% died within 30 days post-hospital discharge period.

**Fig. 2:**
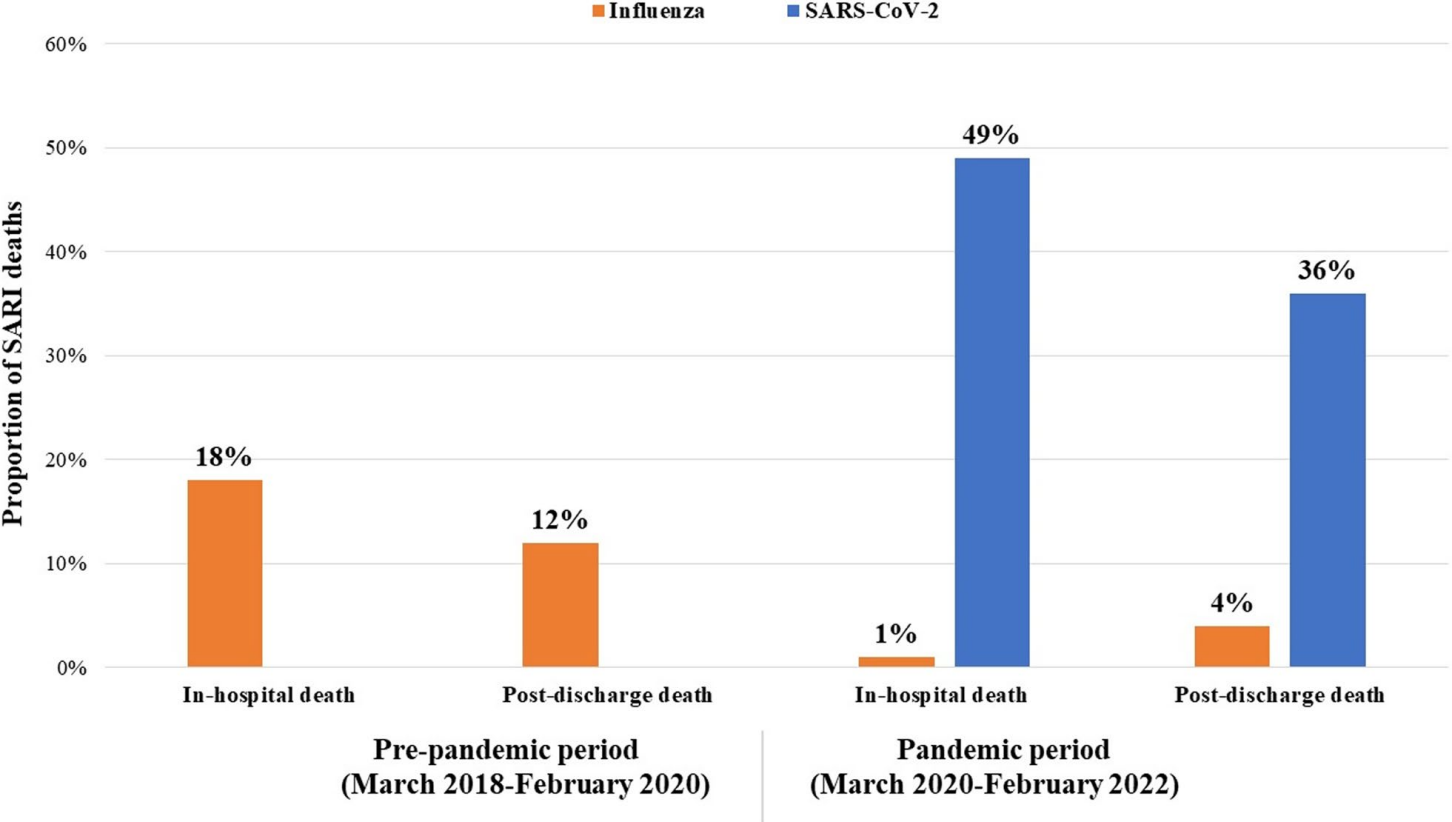
Influenza and SARS-CoV-2 virus detected among in-hospital and post-discharge SARI death cases during the pre- and COVID-19 pandemic periods

**Fig. 3:**
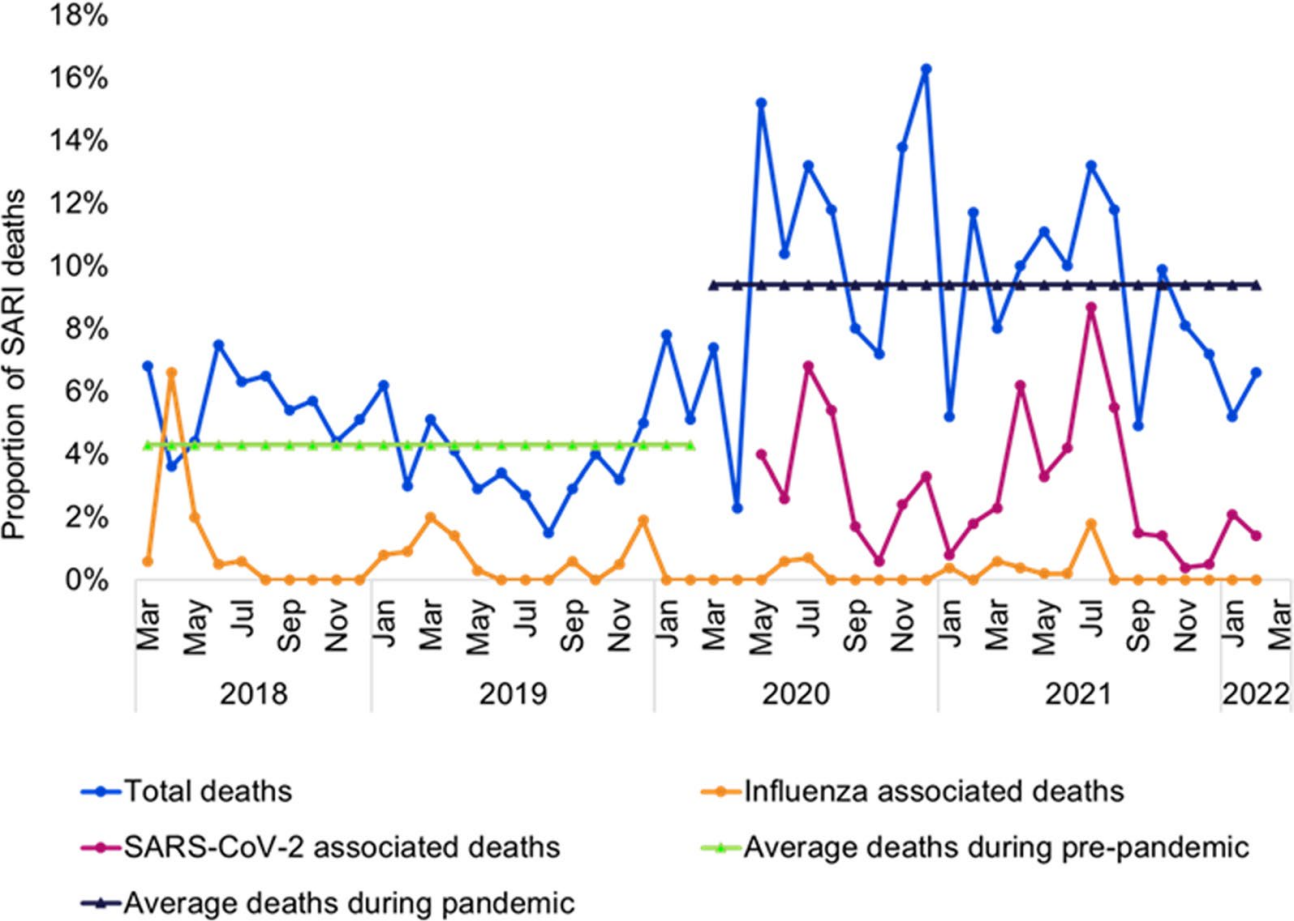
Monthly distribution of deaths among SARI patients during the pre- and COVID-19 pandemic periods

During the COVID-19 pandemic period, patients were tested for both influenza and SARS-CoV-2 virus. Among the 521 recorded SARI deaths, influenza virus was detected in only 2.3% (12) of patients, whereas SARS-CoV-2 was detected in 41.8% (218) of them. Additionally, co-infection of both viruses was identified in only 1 (0.2%) SARI patient, who died during this pandemic period. Influenza virus was detected in 1% of in-hospital death cases and 4% of post-discharge deaths, whereas SARS-CoV-2 was detected in 49% of the in-hospital death cases and 36% post-discharge death cases (Fig. 2). In the pre-pandemic period, an upsurge of influenza-associated SARI deaths was commonly observed from March to July, whereas in the pandemic period, SARS-CoV-2 was the predominant virus responsible for SARI deaths in these months (Fig. 3).

### Factors associated with deaths of SARI patients in the pre-pandemic and pandemic periods

During the pre-COVID-19 pandemic period, after adjusting for age and smoking status, SARI patients who had a history of one or more co-morbid conditions had 1.33 times (95% CI: 1.01–1.73) of higher risk of dying than those without any co-morbid conditions. After adjusting for age, smoking status and co-morbid conditions, adults who experienced difficulty breathing during admission had a 2.36-fold (95% CI: 1.65–3.36) increased risk of dying during this period compared to those who did not have such symptoms (Table 2).

**Table 2:**
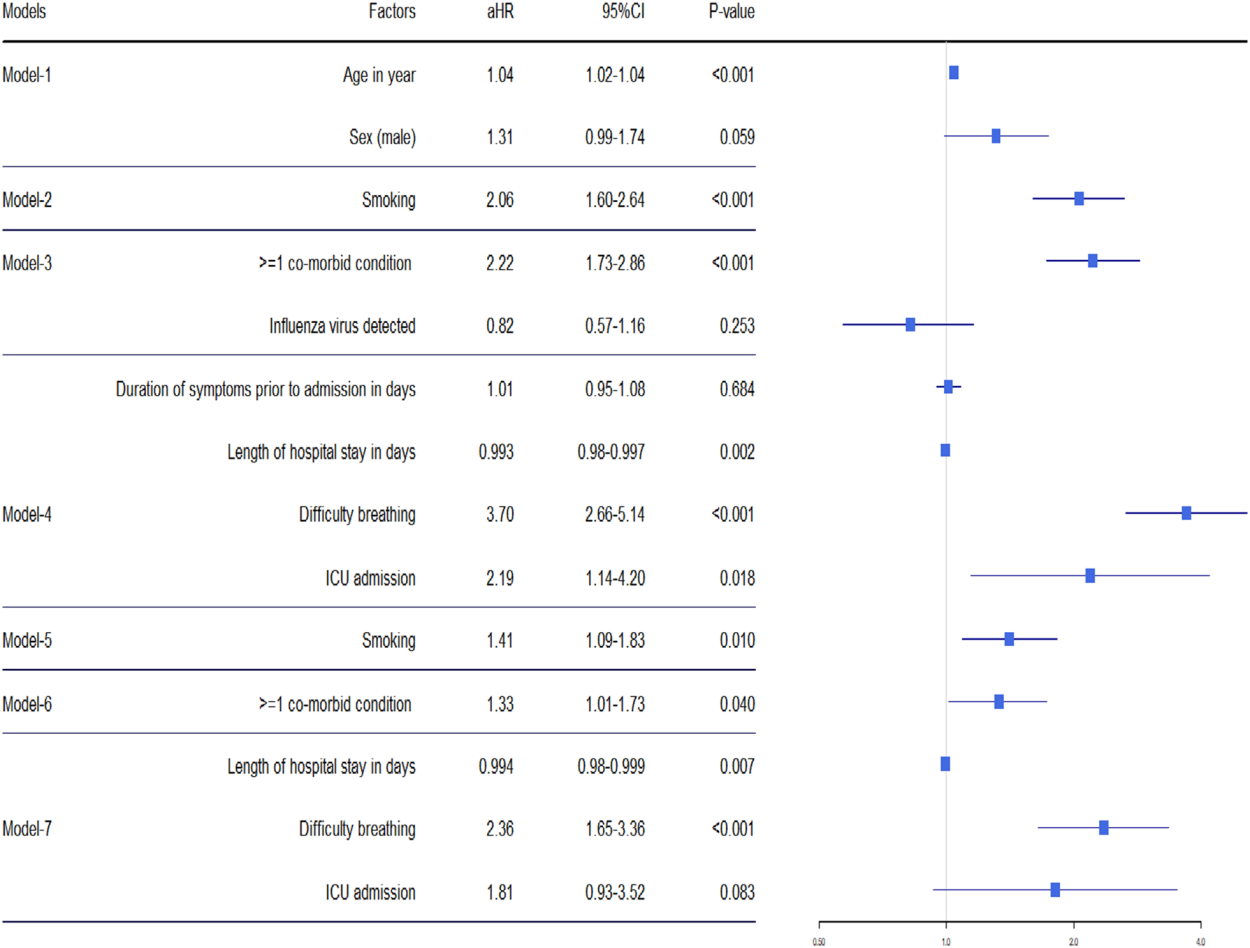
Factors associated with SARI deaths in adults enrolled during the pre-COVID-19 pandemic period (*N* = 264)

In models 1–4, the direct relationship between outcome and explanatory variables/potential predictors of each level was presented without adjusting the variables from other levels. Model 5 included the smoking variable along with the age variable as covariate. Model 6 included at least one co-morbid condition with the covariates smoking, and age variables. Model 7 included intensive care unit (ICU) admission, difficulty breathing, and length of hospital stay in days variables, along with the covariates at least one co-morbid conditions, age, and smoking variables

During the COVID-19 pandemic period, deaths from SARI were associated with the presence of the SARS-CoV-2 virus. After accounting for age, sex and smoking status, SARI patients who tested positive for SARS-CoV-2 exhibited a 1.26-fold increased risk of mortality (95% CI: 1.05–1.51) compared to those who tested negative for SARS-CoV-2. Furthermore, SARI patients who experienced respiratory difficulties upon admission had a significantly increased risk of death, with a hazard ratio of 3.01 (95% CI 1.57–3.35). This finding remained consistent even after adjusting for both age, sex and the detection of influenza and SARS-CoV-2 viruses and such SARI patients with breathing difficulty had 2.30 times increased risk of dying during the pandemic (Table 3).

**Table 3:**
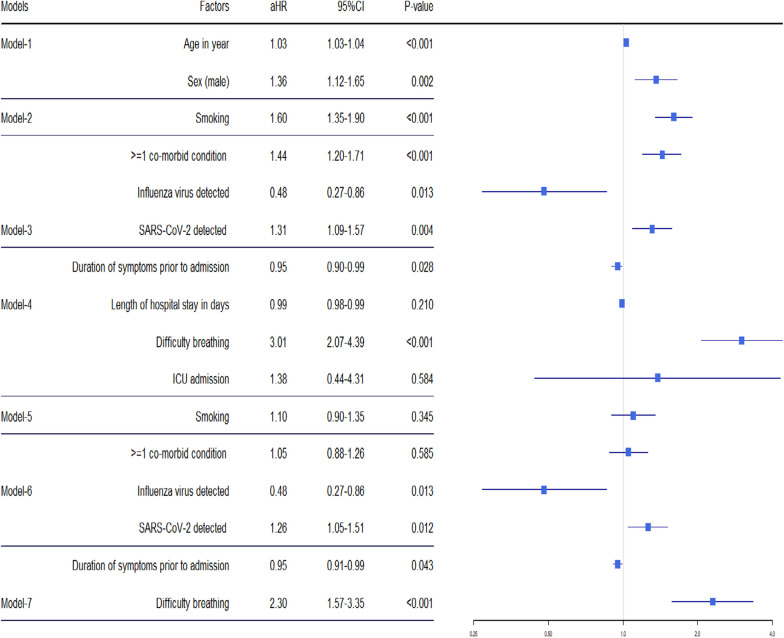
Factors associated with SARI deaths in adults enrolled during the COVID-19 pandemic period (*N* = 521)

In models 1–4, the direct relationship between outcome and explanatory variables/potential predictors of each level was presented without adjusting the variables from other levels. Model 5 included the smoking variable along with age and sex variable as covariate. Model 6 included at least one co-morbid conditions, influenza and SARS-CoV-2 virus detected with the covariates age and sex variables. Model 7 included duration of symptom prior to admission in days and length of hospital stay in days variables, along with the covariates age, sex, influenza virus and SARS-CoV-2 detected variables

## Discussion

The COVID-19 pandemic resulted in a marked escalation of SARI cases and fatalities worldwide, attributed chiefly to the emergence and propagation of the SARS-CoV-2 virus. Our analysis underscores this trend in Bangladesh, where SARI mortality doubled during the pandemic, surging to 13.62% from the pre-pandemic rate of 6.01%. Notably, over one-third of observed SARI deaths were linked to SARS-CoV-2 detection, underscoring the substantial mortality risk posed by the virus among SARI patients. This trend parallels findings from an Indian study involving 500 hospitalized SARI patients, indicating a higher mortality rate in COVID-19-positive SARI cases (34.1%) compared to COVID-negative instances (20.1%) [19]. Other studies also demonstrated that SARI patients had three times more risk of death due to COVID-19 during the pandemic than other respiratory viruses [24, 25]. These collective insights imply that the heightened virulence of SARS-CoV-2, coupled with limited interventions during the pandemic, likely contributed to adverse outcomes among SARI patients.

In the pre-pandemic period, influenza accounted for 18% of in-hospital SARI fatalities in this study, a figure that markedly declined to 1% during the pandemic. Conversely, SARS-CoV-2 stood out as the sole cause of 49% of hospital-admitted SARI deaths during this time. This aligns with other LMIC, middle-income and developed countries as India, the USA, Belgium, France, and Austria, revealing a heightened risk of in-hospital mortality (up to five times) among COVID-19 patients compared to influenza cases [19, 26–30]. Consequently, global influenza cases and deaths saw a notable decline during the pandemic, with minimal influenza activity and associated hospitalizations during the 2020–2021 flu seasons [31–33].

Bangladesh similarly experienced a milder flu season in 2020, lasting just five weeks compared to the average 12.5 weeks of preceding seasons [34]. This reduction of influenza circulation could be attributed to multifaceted measures enacted by the government to curb SARS-CoV-2 spread, including mandated mask-wearing, social distancing, hygiene protocols, and closures of educational and transportation facilities, along with curtailment of public gatherings [35]. Nevertheless, with easing restrictions, the resurgence of influenza strains remains a concern, underscoring the importance of preparedness to mitigate its potential resurgence and associated impact on spread and mortality.

Our study revealed that over half of SARI deaths, both before and during the pandemic, occurred after hospital discharge, even among those with partial or full recovery. A systematic review of 91 articles focusing on post-discharge COVID-19 patient outcomes exhibited a 7.9% all-cause mortality rate within 30 days of discharge [36]. In a retrospective cohort study from the UK, over 1 in 10 acute COVID-19 patients succumbed to post-discharge deaths [37]. One of the possible explanations for this increased post-discharge SARI-associated deaths in our study can be re-infection with the same virus or new infection. Additionally, premature discharge without full recovery at the patients/family members request might also have led to the increased number of post-discharge death in our study. Results from the Matlab Demographic and Health Surveillance in Bangladesh found that the most common reason for requesting early discharge were the family members' perception that the patient's health would not improve (52%), perceived health improvement (8.7%), or misjudgment of illness severity (4.3%) [38]. Notably, data on the 30-day post-discharge mortality of COVID-19-positive SARI patients remain limited. This underscores the imperative of sustained monitoring and follow-up care for SARI patients after hospital discharge in resource constrained settings. Further research in larger cohorts is essential to discern potential risk factors and interventions to mitigate post-discharge SARI deaths.

This study highlights significant risk factors for SARI patient’s mortality before and during the pandemic. Notably, respiratory distress on admission correlated with higher mortality rates in both time periods and such SARI patients had more than twofold increased risk than those who did not experience breathing difficulty during their illness period. We have also found that among adults, smoking history, and co-morbidities were independent risk factors of pre-pandemic SARI mortality as have also been reported in other studies [39–42]. However, these factors were not associated with SARI mortality during the pandemic period in our analysis. Similarly, we did not find an association of ICU admission with SARI deaths in both pre- and post-pandemic periods, likely due to a limited number of patients (9 during the pandemic and 19 pre-pandemic), as the study hospitals lacked ICU facilities or had only a few ICU beds, leading to many patients being treated in general wards even if they required ICU admission.

During the pandemic period, the most important predictors of SARI deaths was the presence of the SARS-CoV-2 virus. After accounting for age, sex and smoking status, SARI patients who tested positive for SARS-CoV-2 exhibited a 1.26-fold increased risk of mortality compared to those who tested negative for SARS-CoV-2, thus further emphasizing the necessity of comprehensive immunization strategies, particularly for completing the scheduled doses of SARS-CoV-2 vaccination and promoting booster uptake to reduce SARI deaths in this population.

While this study draws upon data from both pre-pandemic and pandemic adult SARI patients, we acknowledge certain limitations. Firstly, our exclusive focus on testing for influenza and SARS-CoV-2 may have constrained our understanding of other potential causative agents contributing to SARI-related fatalities among these patients. Secondly, our analysis encompasses only those seeking and subsequently admitted to hospital care, potentially overlooking cases of influenza-like illnesses managed in outpatient settings. Thirdly, our 30-day post-discharge follow-up relied on self-reported patient deaths from family members, omitting the use of verbal autopsies, which could help mitigate potential bias in ascertaining the precise cause of death. Next, despite BMI being an important risk factor leading to severity and adverse outcomes in COVID-19 patients, our study could not incorporate it in the risk factor analysis of pandemic SARI deaths as the routine surveillance system did not record data on the BMI, nutritional status, or anthropometric measurements of the participants. Similarly, the surveillance did not capture data on disease severity such as severity scores or measure oxygen saturation level or partial pressure of oxygen in blood for enrolled SARI patients limiting our ability to include this in our regression analysis. Lastly, our surveillance system operates six days a week, potentially resulting in missed admissions of SARI patients during weekends.

## Conclusion

The COVID-19 pandemic precipitated a surge in SARI-related fatalities primarily attributed to the SARS-CoV-2 virus, while a decline in influenza prevalence and severity coincided, possibly owing to the protective measures against SARS-CoV-2 transmission. The primary factor driving the SARI mortality during pandemic period was SARS-CoV-2 infection thus underscoring the need for comprehensive SARS-CoV-2 vaccination strategies in adults. Furthermore, our findings reveal a pronounced trend of post-discharge mortality pre- and during the pandemic, necessitating measures to forestall premature discharge, ensure robust post-discharge monitoring and nursing care, and prompt further research to uncover factors contributing to post-discharge mortality, thereby strengthening post-discharge surveillance, and ultimately curtailing avoidable losses in SARI patients.

### Supplementary Information

**Additional file 1**: **Figure S1**. Conceptual framework relationship between SARI deaths and explanatory variables. For adults, (a) during the pre-pandemic period, the variables included age, sex, smoking, presence of one or more co-morbid conditions, detection of influenza virus, duration of symptom prior to admission in days, length of hospital stay in days, requiring ICU admission during hospital stay and difficulty breathing on admission. (b) during the pandemic period, we additionally added the variable SARS-CoV-2 detected with all the other variables of the pre-pandemic period in the multivariable analysis.

## Data Availability

All relevant data are provided within the manuscript. According to institutional policy, datasets which were analyzed in this study can be obtained from the icddrb’s research administration upon reasonable request to the corresponding author.

## Abbreviations

SARI: Severe acute respiratory infections
LMIC: Low- and middle-income countries
SARS-CoV-2: Severe Acute Respiratory Syndrome Corona Virus 2
COPD: Chronic obstructive pulmonary disease
HIV: Human Immunodeficiency Virus
COVID-19: Corona virus disease 2019
HBIS: Hospital-Based Influenza Surveillance
WHO: World Health Organization
