# Supplementary figures and images for: Characterizing deaths among adult patients with severe acute respiratory infection: during the pre- and COVID-19 pandemic periods in Bangladesh, 2018–2022
Source: Trop Med Health. 2023 Dec 19;51:70. doi: 10.1186/s41182-023-00565-1 (PMC10729565; doi:10.1186/s41182-023-00565-1)

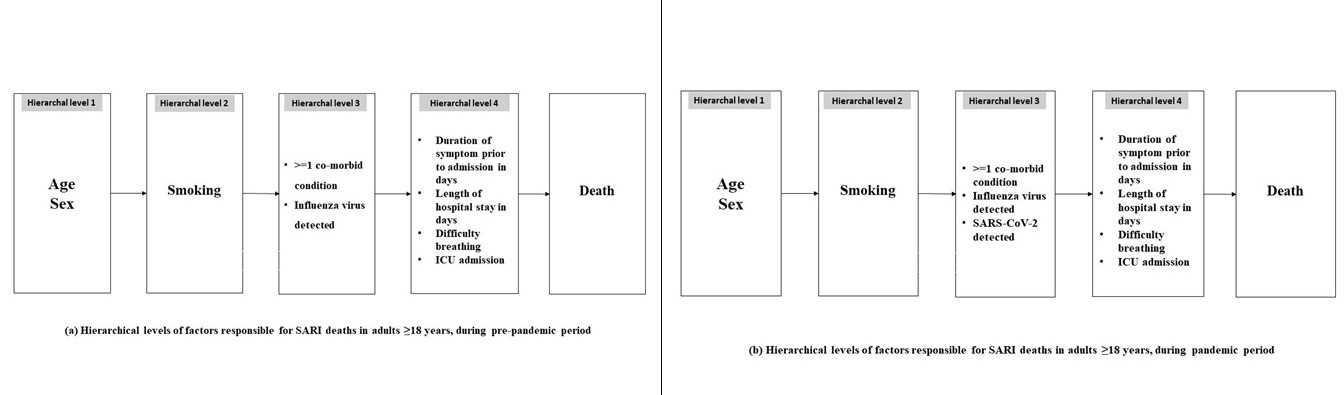

Supplement: Supplementary file 1 — Additional file 1: Figure S1. Conceptual framework relationship between SARI deaths and explanatory variables. For adults, (a) during the pre-pandemic period, the variables included age, sex, smoking, presence of one or more co-morbid conditions, detection of influenza virus, duration of symptom prior to admission in days, length of hospital stay in days, requiring ICU admission during hospital stay and difficulty breathing on admission. (b) during the pandemic period, we additionally added the variable SARS-CoV-2 detected with all the other variables of the pre-pandemic period in the multivariable analysis. [file 41182_2023_565_MOESM1_ESM.jpeg]
